# A Generic Strategy to Generate Bifunctional Two-in-One Antibodies by Chicken Immunization

**DOI:** 10.3389/fimmu.2022.888838

**Published:** 2022-04-11

**Authors:** Julia Harwardt, Jan P. Bogen, Stefania C. Carrara, Michael Ulitzka, Julius Grzeschik, Björn Hock, Harald Kolmar

**Affiliations:** ^1^ Institute for Organic Chemistry and Biochemistry, Technical University of Darmstadt, Darmstadt, Germany; ^2^ Ferring Darmstadt Laboratory, Biologics Technology and Development, Darmstadt, Germany; ^3^ Ferring Biologics Innovation Centre, Biologics Technology and Development, Epalinges, Switzerland; ^4^ Centre for Synthtic Biology, Technical University of Darmstadt, Darmstadt, Germany

**Keywords:** bispecific antibody, two-in-one antibody, dual action fab, yeast display, chicken-derived

## Abstract

Various formats of bispecific antibodies exist, among them Two-in-One antibodies in which each Fab arm can bind to two different antigens. Their IgG-like architecture accounts for low immunogenicity and also circumvents laborious engineering and purification steps to facilitate correct chain pairing. Here we report for the first time the identification of a Two‐in‐One antibody by yeast surface display (YSD) screening of chicken-derived immune libraries. The resulting antibody simultaneously targets the epidermal growth factor receptor (EGFR) and programmed death‐ligand 1 (PD-L1) at the same Fv fragment with two non-overlapping paratopes. The dual action Fab is capable of inhibiting EGFR signaling by binding to dimerization domain II as well as blocking the PD-1/PD-L1 interaction. Furthermore, the Two-in-One antibody demonstrates specific cellular binding properties on EGFR/PD-L1 double positive tumor cells. The presented strategy relies solely on screening of combinational immune-libraries and obviates the need for any additional CDR engineering as described in previous reports. Therefore, this study paves the way for further development of therapeutic antibodies derived from avian immunization with novel and tailor-made binding properties.

## Introduction

In recent years, an increasing number of bispecific antibody (bsAb) approaches have been developed (1, 2). BsAbs, which can simultaneously target two distinct antigens, enabled new therapeutic mechanisms of action that can neither be addressed by conventional monoclonal antibodies (mAbs) nor by their combination (3–5). A subclass of bsAbs are Two-in-One antibodies with dual action Fabs (DAFs), in which each Fab arm addresses two distinct antigens, resulting in a bispecific, tetravalent IgG-like molecule (6, 7). The classical IgG like bispecific antibody setting requires correct heavy chain heterodimerization as well as correct light chain pairing, which statistically results in only 12.5% of correctly assembled molecules (**Supplementary Figure 1**) (8). Two-in-One antibodies, in contrast, consist of two identical heavy and light chains and can be produced without additional engineering of constant chains (9). Therefore, the need to include unnaturally occurring amino acid sequences as found in knob-into-hole antibodies (10) or orthogonal Fab interfaces (11) is circumvented.

The first Two-in-One antibody was generated by Bostrom et al. based on mutagenesis of the light chain complementarity-determining regions (CDRs) of the HER2 specific antibody trastuzumab resulting in HER2 and VEGF binding (12). Subsequently, mutagenesis approaches were used towards the generation of Two-in-One antibodies targeting HER3 and EGFR (13), IL-4 and IL-5 (14), or VEGF and angiopoietin 2 (15). The Two-in-One antibody duligotuzumab, which targets HER3 and EGFR (13), has been tested in clinical trials for treating epithelial-derived cancer (16, 17), highlighting the importance of this class of therapeutics. However, these antibodies all exhibit partially overlapping CDR residues leading to antigen 1 blocking the binding of antigen 2, consequently allowing binding of only one antigen at the same time.

DutaFabs, in contrast, comprise two independent binding sites within the CDR loops. The H-side paratope consists of CDR H1, H3 and L2, while the L-side paratope comprises CDR L1, L3 and H2. Therefore, these Fabs are able to target two antigens simultaneously with the same Fv region, however the design of DutaFabs is comparatively complex (18). Furthermore, tetravalent IgG-like bispecific constructs were described that do not consist of regular Fab arms but rather of engineered arms in which one VH domain is attached to each of the constant CH_1_ and CL domains (19). Here, one VH is placed at its usual position and the second VH replaces the VL domain in a conventional IgG. It was found that the tetra-VH IgGs can simultaneously bind two antigens on each arm of the IgG molecule (19).

Due to their ability to cross-link receptors, mediate proximity between immune effector cells and tumor cells, or block two disease-related signaling pathways, bsAbs are exceptional therapeutic entities for cancer treatment (20–22). Tumor-specificity of bsAbs can be elevated by simultaneous targeting of two cancer-specific antigens on the same malignant cell (23). Two therapeutic targets being upregulated in many solid tumors are the programmed death ligand 1 (PD-L1) and human epidermal growth factor receptor (EGFR) (24, 25). Overexpression of PD-L1 is observed in a variety of malignancies and represents a mechanism by which cancer evades immune surveillance (24, 26). EGFR, which is natively expressed on epithelial cells in the skin and lung, is overexpressed in a wide range of cancers including bladder cancer, lung cancer, colorectal cancer, and breast cancer, where it is involved in tumor progression and metastasis (25, 27–29). Koopmans and coworkers demonstrated that tumor-specificity can be increased by EGFR directed PD-L1 blockade, resulting in a potentially favorable safety profile of the described bsAb (30).

Most approved therapeutic mAbs were generated by immunization of rodents, including mice, rabbits, or other mammalian species (31). However, due to their close phylogenetic relationship to humans, targeting epitopes which are broadly conserved in mammalian species is challenging. Chicken immunization, in contrast, may result in antibodies targeting epitopes that are not accessible by immunization of mammals (32, 33). Additionally, library generation can be done with a single set of primers because of the gene diversification in birds, significantly reducing the hands-on time and costs compared to rodents (34). Recently, our group described the isolation of highly affine chicken-derived antibodies using yeast surface display (YSD) in combination with fluorescence-activated cell sorting (FACS) (34–36).

**Figure 1:**
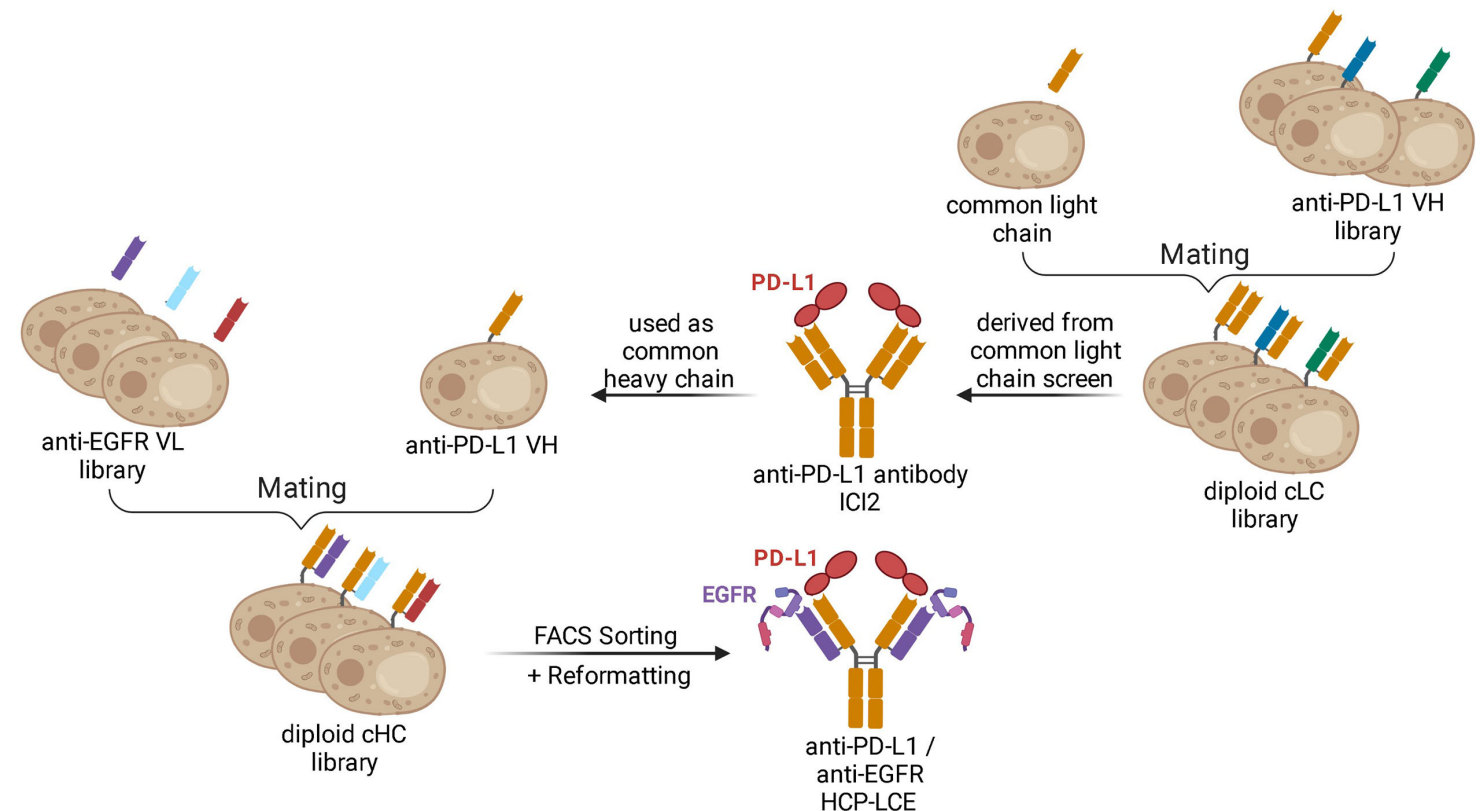
Schematic representation demonstrating the generation of the Two-in-One antibody HCP-LCE. The anti-PD-L1 antibody ICI2 is derived from a common light chain YSD library. The VH fragment of the anti-PD-L1 antibody ICI2 was paired with an anti-EGFR VL library by yeast mating. FACS screening and subsequent reformatting into the full-length antibody format enabled the isolation and production of a Two‐in‐One antibody targeting PD‐L1 and EGFR. Created with BioRender.com.

In this study, we describe the isolation and characterization of the first Two-in-One antibody that simultaneously targets PD-L1 and EGFR with two independent paratopes on a single Fab. It is derived from immunized chickens by combining the heavy chain of a common light chain antibody with an immune light chain library without engineering the antibodies’ CDR regions (**Figure 1**). The Two-in-One antibody demonstrated specific cellular binding properties on EGFR- and PD-L1-expressing tumor cells, as well as inhibition of EGFR-dependent signal transduction and blockage of the PD-1/PD-L1 interaction.

## Results

### Two-in-One Library Generation and Screening

In order to generate a Two-in-One antibody, we sought to combine the heavy chain of a chicken-derived antibody with a chicken-derived immune YSD light chain library followed by subsequent screening for binding properties towards two antigens. As model targets, the extracellular domains of PD-L1 (PD-L1-ECD) and EGFR (EGFR‐ECD) were chosen. Currently, various monoclonal antibodies targeting either EGFR, including the therapeutic antibodies panitumumab (37), necitumumab (38), nimotuzumab (39) and cetuximab (40), or PD-L1, among them durvalumab (41), avelumab (42) and atezolizumab (43) are approved for tumor treatment in multiple countries. Recently, our group isolated a chicken-derived anti‐PD‐L1 antibody called ICI2 (44). Since ICI2 exhibited a common light chain as it was utilized in a multispecific setup, we assumed that the heavy chain CDRs were mainly responsible for antigen recognition and could tolerate various light chains, as reported for other antibodies (45, 46). In order to isolate a Two-in-One antibody targeting both PD-L1 and EGFR, the ICI2 heavy chain was paired with a diversity of anti‐EGFR light chains (**Figure 1**). The light chain library was generated by amplification of VL genes from cDNA derived from a chicken immunized with EGFR-ECD and subsequent insertion into a pYD_1_‐derived vector encoding a human lambda CL by homologous recombination in BJ5464 yeast as previously described (44). The light chain diversity of 2.9x10^8^ transformants was combined with EBY100 yeast cells encoding the ICI2 VH-CH_1_ fragment by yeast mating (**Figure 1**), resulting in adequate oversampling of the estimated light chain diversity, which was estimated to be about 5x10^8^ unique variants.

This diploid common heavy chain yeast library was screened by FACS over three consecutive sorting rounds with 250 nM EGFR‐Fc (**Supplementary Figure 2A**). This resulted in the enrichment of a yeast population carrying the genes for Fab fragments recognizing both EGFR‐Fc and PD‐L1‐Fc with 250 nM of the respective antigen. Fc binding could be excluded based on binding analysis to an unrelated Fc fusion protein (**Supplementary Figure 2B**). Sequence analysis of ten randomly chosen clones revealed one distinct VL sequence, which was enriched in the sorting process.

### EGFR Epitope Mapping on the Subdomain Level

Expi293F cells were co-transfected using the isolated VL sequence reformatted into a pTT5-derived vector encoding a lambda CL sequence and a pTT5 vector encoding the ICI2 heavy chain as described before (44, 47). Purification of the Two-in-One antibody, hereafter referred to as HCP-LCE (heavy chain PD-L1 – light chain EGFR), was performed using Protein A affinity chromatography.

The extracellular region of EGFR consists of two homologous ligand-binding domains (domains I and III) and two cystine-rich domains (domains II and IV) (48). The binding of EGF to the EGFR monomers at domains I and III promotes domain rearrangement to expose the dimerization arm in domain II finally resulting in the generation of EGFR homodimers (49, 50). For full EGFR activation, ligand binding and EGFR dimerization are crucial (27).

**Figure 2:**
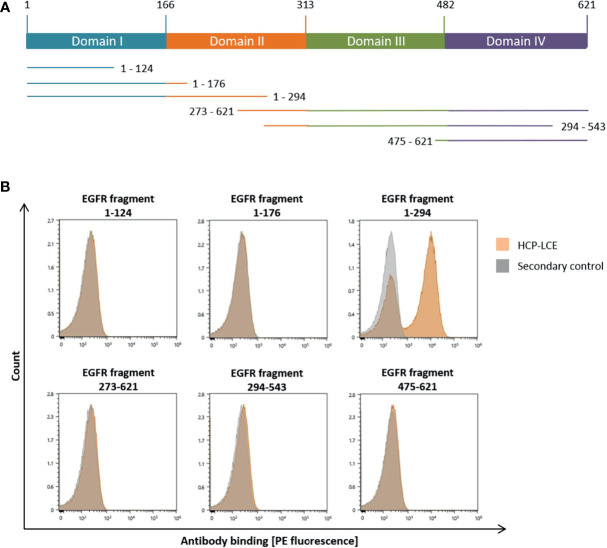
YSD-based EGFR epitope mapping of HCP-LCE. **(A)** Schematic representation of EGFR extracellular domains I to IV and the six EGFR fragments investigated in this study. **(B)** Binding of HCP‐LCE (orange) to yeast cells expressing different truncated EGFR fragments was detected using the goat anti-human Fc PE antibody. Measurements without HCP-LCE (grey) served as a negative control. HCP-LCE binds to EGFR fragment 1-294.

To analyze which of the four extracellular EGFR domains HCP-LCE targeted, flow cytometric analysis was performed using yeast cells displaying truncated fragments of the EGFR-ECD, as described previously (51, 52) (**Figure 2A**). Since HCP-LCE exclusively targets EGFR fragment 1-294 but neither 1-124 nor 1-176, it was mapped to EGFR domain II, which is involved in receptor dimerization (**Figure 2B**). Cetuximab is known to target domain III inhibiting EGF-binding to EGFR, while matuzumab blocks the receptor activation by sterically preventing the domain rearrangement (53). Binding of HCP-LCE to EGFR domain II may suggest inhibition of receptor dimerization and subsequent activation.

### Affinity Measurement

Biolayer interferometry (BLI) measurements were performed in order to determine the affinity of HCP-LCE to both PD‐L1 and EGFR. In order to confirm that non-overlapping paratopes were present and binding to both antigens was possible with a single Fab, an additional one-armed HCP-LCE variant was produced using Knob‐into‐Hole (KiH) technology, as previously described (10). Furthermore, affinity measurements of the HCP‐LCE heavy chain combined with an unrelated light chain (ICI2_H2) and that of the HCP‐LCE light chain together with an unrelated heavy chain (LCE) to the two proteins of interest were performed.

**Figure 3:**
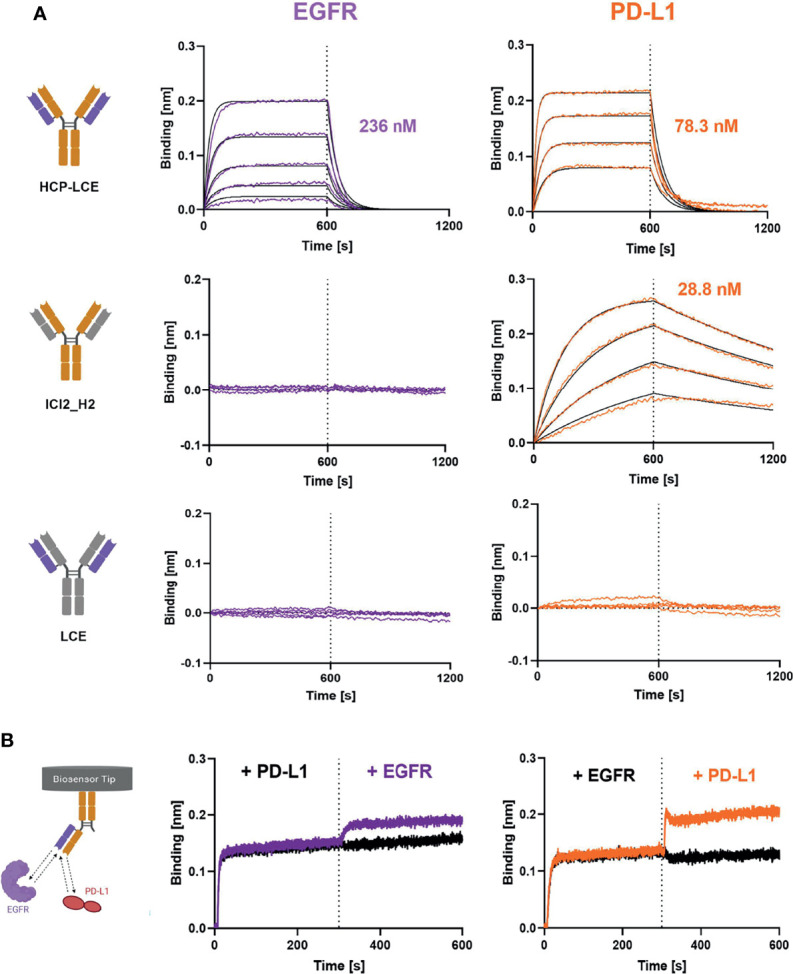
Characterization of antigen binding of the Two-in-One antibody HCP-LCE by BLI-measurements. **(A)** BLI-measurements of HCP-LCE, ICI2_H2 and LCE against EGFR and PD-L1. HCP-LCE binds both antigens, whereas ICI2_H2 exclusively targets PD-L1. LCE shows no binding to either antigen. **(B)** BLI-assisted simultaneous binding assay. The one-armed HCP-LCE variant was loaded onto biosensors and antigens are added step-wise, revealing simultaneous EGFR and PD-L1 binding. Created with BioRender.com.

HCP-LCE was able to bind both antigens with a K_D_ of 78.3 nM and 236 nM for PD-L1 and EGFR binding, respectively, exhibiting a high off-rate (**Figure 3A**, **Table 1**). The one-armed variant showed slightly lower affinities to both antigens which might presumably be caused by the lower avidity (**Supplementary Figure 3**). Variant ICI2_H2 exclusively targeted PD-L1 with an affinity in the double digit nanomolar range, whereas variant LCE showed no binding to either PD-L1 or EGFR (**Figure 3A**, **Table 1**). This suggests that only the three HCP-LCE heavy chain CDRs are responsible for PD-L1 binding, contrary to EGFR binding involving overlapping heavy and light chain CDRs.

**Table 1:** Biophysical properties of HCP-LCE, oaHCP-LCE, LCE, ICI2_H2 and SEB7xICI2_H2 including affinity, kinetic binding rates, melting temperature and aggregation.

Antibody	K_D_ [nM]		k_on_ [M^-1^ s^-1^]		k_dis_ [s^-1^]		T_M_ [°C]	Aggregation [%]
	EGFR	PD-L1	EGFR	PD-L1	EGFR	PD-L1		
HCP-LCE	236 ± 10.7	78.3 ± 1.36	4.15 × 10^5^ ± 1.58 × 10^4^	9.73 × 10^5^ ± 1.37 × 10^4^	9.81 × 10^‐2^ ± 2.41 × 10^‐3^	7.62 × 10^‐2^ ± 7.78 × 10^‐4^	58.9	2.22
oaHCP-LCE	295 ± 17.1	117 ± 4.15	3.88 × 10^5^ ± 1.92 × 10^4^	9.29 × 10^5^ ± 2.62 × 10^4^	1.14 × 10^‐1^ ± 3.52 × 10^‐3^	1.09 × 10^‐1^ ± 2.32 × 10^‐3^	59.6	12.44
LCE	–	–	–	–	–	–	61.1	1.27
ICI2_H2	–	28.8 ± 0.236	–	1.22 × 10^5^ ± 5.46 × 10^2^	–	3.5 × 10^‐3^ ± 2.40 × 10^‐5^	63.0	0
SEB7xICI2_H2	7.85 ± 0.173	29.9 ± 0.556	2.34 × 10^5^ ± 1.56 × 10^3^	1.30 × 10^5^ ± 1.41 × 10^3^	1.84 × 10^‐3^ ± 3.87 × 10^‐5^	3.90 × 10^‐3^ ± 5.90 × 10^‐5^	65.8	10.8

Since EGFR and PD-L1 are widely expressed on healthy cells (30, 54), simultaneous binding of both proteins is essential to increase tumor-specificity. To analyze the binding behaviour of HCP-LCE and to verify whether both antigens can be targeted simultaneously with a single Fab, oaHCP-LCE was loaded onto AHC biosensors and sequentially incubated with both target proteins of interest. Here it was essential to use the one-armed variant, since the symmetric HCP-LCE antibody could target one antigen with each Fab arm. Binding to PD-L1 first and EGFR second as well as reverse binding was considered. The oaHCP-LCE variant was able to bind both antigens simultaneously, regardless of the order of target protein incubation (**Figure 3B**). These findings indicate that the paratopes do not overlap.

### Biophysical Characterization

Size-exclusion chromatography (SEC) profiles demonstrated that HCP-LCE exhibited favorable properties with almost no measurable aggregation (**Table 1**, **Supplementary Figure 5**). Monospecific antibodies ICI2_H2 and LCE showed an excellent aggregation profile, while the bispecific EGFR- and PD-L1-binding antibody SEB7xICI2_H2 and the oaHCP-LCE variant exhibited aggregation of 10.8% and 11.20%, respectively (**Table 1**). SEB7 has already been characterized previously (52). Retention times were as expected (**Table 1**, **Supplementary Figure 5**), indicating the accurate size of the antibodies produced, which is in accordance with SDS-PAGE analysis (**Supplementary Figure 4**). In the case of both antibodies with KiH, oaHCP-LCE and SEB7xICI2_H2, SDS-PAGE demonstrated the expression of similar amounts of both heavy chains, with the TwinStrep-tagged Fc having a significantly higher molecular weight than the His-tagged Fc (**Supplementary Figure 4**).

Thermal stability was analyzed using NanoDSF, yielding T_M_ values between 58.9°C and 67.3°C, indicating high thermal stability of all variants (**Table 1**, **Supplementary Figure 5**). For full-length antibodies, two to three T_M_ values are expected based on unfolding of the Fab fragment and CH_2_/CH_3_ domains (55). The lowest T_M_ value was utilized to compare the stability of all antibodies generated.

### EGF and PD-1 Competition

**Figure 4:**
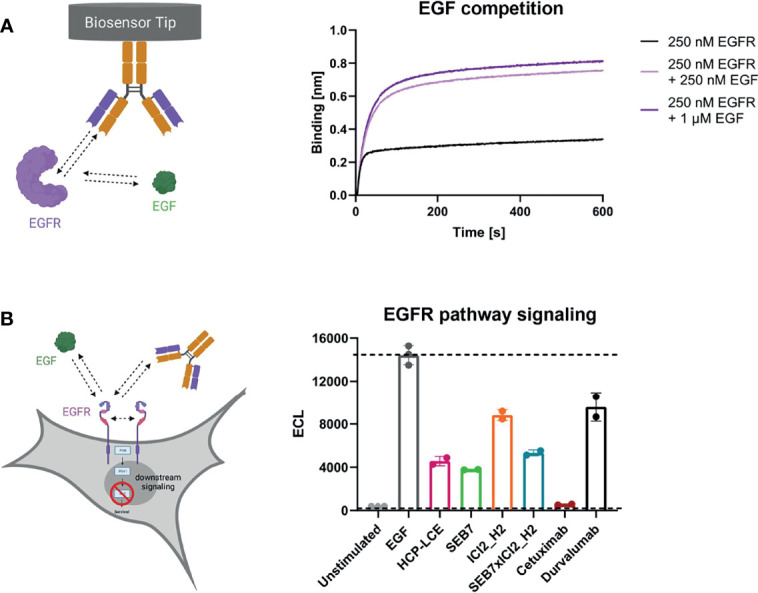
EGF competition and EGFR signaling assays. **(A)** BLI-assisted EGF competition assay. HCP-LCE was loaded onto AHC biosensors and subsequently associated to EGFR pre‐incubated with varying EGF concentrations. HCP-LCE binds to EGFR despite EGF binding. **(B)** Cell-based EGFR signaling assay. EGF-induced AKT phosphorylation was analysed in EGFR-positive A549 cells. SEB7 (green), ICI2_H2 (orange), the bispecific construct SEB7xICI2_H2 (blue), cetuximab (red) and durvalumab (black) were tested in comparison to the Two-in-One antibody HCP-LCE (pink). All measurements were performed in triplicates. Created with BioRender.com.

To investigate antibody-mediated ligand receptor blockade, a competition assay was performed by aid of biolayer interferometry. For analysis of EGF competition, anti-human IgG Fc Capture (AHC) biosensors were loaded with HCP-LCE and subsequently associated to 250 nM EGFR pre-incubated with 250 nM or 1000 nM EGF. Due to the binding of EGFR to EGF, the complex exhibits a larger molecular size compared to EGFR alone. Binding of HCP-LCE to this complex, therefore, results in a higher increase in layer thickness compared to binding of EGFR alone (**Figure 4A**), indicating that the antibody does not target the interaction site of EGF and EGFR, which is consistent with the YSD-based epitope mapping experiment (**Figure 2B**). EGF binds simultaneously to EGFR domains I and III, whereas HCP-LCE targets EGFR domain II, which is involved in receptor dimerization (48).

For total EGFR activation, ligand binding and EGFR dimerization are essential. EGF binding to EGFR promotes domain rearrangement to expose the dimerization arm in domain II (49). Since HCP-LCE targets domain II, it was investigated whether the Two-in-One antibody is able to inhibit EGF-induced EGFR dimerization by measuring the downstream phosphorylation of AKT in EGFR-positive A549 cells. In the presence of HCP-LCE (100 µg/mL), AKT phosphorylation is significantly reduced compared to the EGF stimulated control (20 ng/mL) (**Figure 4B**). Anti-EGFR antibody SEB7 and SEB7xICI2_H2, which also target EGFR domain II (52), showed comparable inhibition of AKT phosphorylation as HCP-LCE. The EGFR domain III binder Cetuximab (56) completely inhibited EGF-induced phosphorylation of AKT (**Figure 4B**) since binding of domain III blocked EGF binding (52). To conclude, EGFR signaling is significantly inhibited by HCP-LCE binding to dimerization domain II without interfering EGF-binding to its receptor.

**Figure 5:**
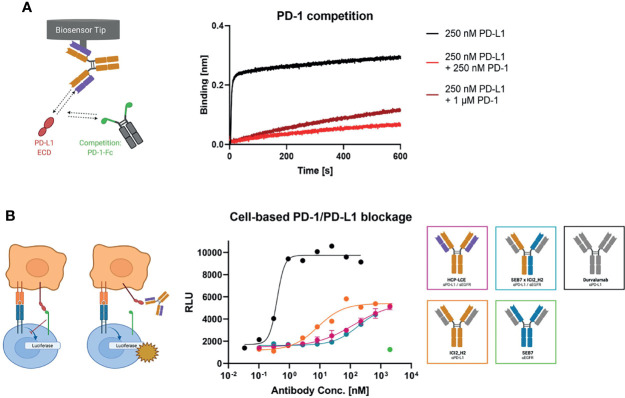
PD-1 competition and PD-1/PD-L1 blockage assays. **(A)** BLI-assisted PD-1 competition assay. HCP-LCE was loaded onto FAB2G biosensors and subsequently associated to PD-L1 pre‐incubated with varying PD-1 concentrations. The binding of HCP-LCE to PD-L1 at different PD‐1 concentrations reveals dose-dependent binding. **(B)** Cell-based PD-1/PD-L1 blockage assay. SEB7 (green), ICI2_H2 (orange), the bispecific construct SEB7xICI2_H2 (blue) and durvalumab (black) were tested in comparison to the Two-in-One antibody HCP-LCE (pink). EC_50_ values: durvalumab, 0.39 nM; ICI2_H2, 8.60 nM; SEB7xICI2_H2, 179 nM; HCP-LCE, 214 nM. Luciferase activity is plotted against the logarithmic antibody concentration. All measurements were performed in duplicates, and the experiments were repeated at least three times, yielding similar results. Created with BioRender.com.

To investigate HCP-LCE-mediated PD-1/PD-L1 competition, HCP-LCE was loaded onto FAB2G biosensors and was associated to 250 nM PD-L1 pre-incubated with either 250 nM or 1000 nM of PD-1. HCP-LCE exhibited significantly impaired binding to PD-L1 in the presence of PD-1, indicating that the antibody targets and blocks the PD-1/PD-L1 interaction site (57) (**Figure 5A**). This antibody-mediated PD-1/PD-L1 blockade was expected, as the heavy chain of HCP-LCE is derived from the anti-PD-L1 antibody ICI2 described by Bogen and coworkers, which demonstrated blockage of the PD-1/PD-L1 axis (44).

Verification of the PD-L1 blockage activity of HCP-LCE was performed in a cell-based context using the Promega PD-L1 blockade assay kit. HCP-LCE showed notable PD-L1 blockade, although with a less dominant effect compared with ICI2_H2 (**Figure 5B**). This diminished EC_50_ value most probably originates from the lower affinity towards PD-L1 binding. However, in combination with the original common light chain dFEB4-1, the ICI2 heavy chain exhibited a blockage of the PD-1/PD-L1 interaction comparable to durvalumab, as well as a significantly higher affinity PD-L1 binding (44). SEB7xICI2_H2 exhibited comparable PD-L1 blockade as HCP-LCE, despite monovalent target binding of the bispecific antibody. The anti-EGFR antibody SEB7 did not interfere with PD-1/PD-L1 interaction even at high concentrations (**Figure 5B**). Conclusively, the binding of PD-1 to PD-L1 is significantly inhibited by HCP-LCE, indicating its function as a checkpoint inhibitor.

### Cell Titration on A431 Cells

**Figure 6:**
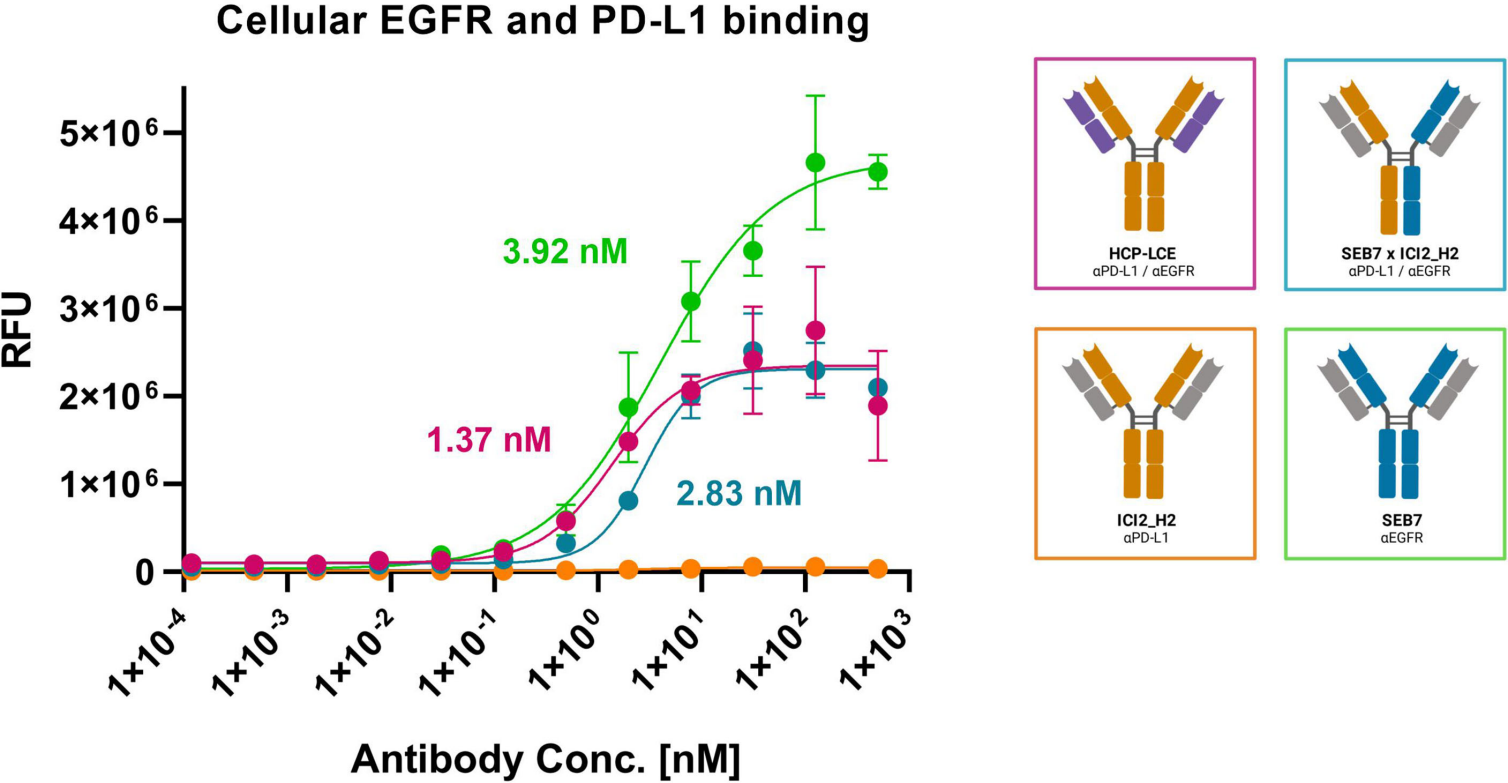
Cell binding of EGFR and PD-L1 on A431 cells. Cell titration of HCP-LCE (pink), ICI2_H2 (orange), SEB7 (green) and the bispecific construct (blue) on EGFR/PD-L1 double positive A431 cells. A variable slope four-parameter fit was utilized to fit the resulting curves. EC_50_ values: HCP-LCE, 1.37 nM; SEB7, 3.92 nM; SEB7xICI2_H2, 2.83 nM. All measurements were performed in triplicates, and the experiments were repeated at least three times, yielding similar results.

Since HCP-LCE is expected to provide increased tumor-specificity compared to the bispecific antibody SEB7xICI2_H2 and the corresponding monospecific antibodies due to simultaneous binding of EGFR and PD-L1 at each Fab arm, cell binding experiments were performed on EGFR/PD-L1 double-positive A431 cells by flow cytometry. Cells were stained with the respective antibody at a concentration ranging from 0.12 pM to 500 nM utilizing a four-fold dilution series and binding was verified using an anti-human Fc PE detection antibody. HCP-LCE exhibited cellular binding with an EC_50_ value of 1.37 nM, while the EC_50_ value of the bsAb SEB7xICI2_H2 was comparable (**Figure 6**). EGFR overexpression on cancer cells typically exceeds that of PD-L1 (44), as demonstrated by A431 binding of the monospecific antibodies (**Figure 6**; **Supplementary Figure 6A**). The antibodies did not show binding to EGFR/PD-L1 double-negative HEK cells, excluding non-specific cell binding (**Supplementary Figure 6**). These data indicate that simultaneous binding of EGFR and PD-L1 at both Fab arms enhances A431 cell binding, compared to monovalent or bivalent target protein binding.

## Discussion

Most antibodies utilize the heavy chain CDRs as the dominant moiety in antigen binding and can tolerate some mutations at the light chain CDRs (45, 46). This property was exploited to isolate the first Two-in-One antibody from a phage display library by mutating the light chain CDR fragments (12, 58). Subsequently, further engineering approaches were used for the generation of Two-in-One antibodies involving computational-based design, structural-guided design or random mutagenesis (13–15, 18, 19).

In this study, we generated the first chicken-derived Two-in-One antibody without CDR engineering that simultaneously targets EGFR and PD-L1 within the same Fv region. To this end, we paired the heavy chain of the chicken-derived anti-PD-L1 antibody ICI2 (44) with a chicken-derived anti-EGFR light chain immune library by yeast mating. Isolation of the Two-in-One antibody HCP-LCE was performed by three rounds of FACS-based selection using YSD. HCP-LCE showed binding affinities in the double- to triple-digit nanomolar range, favorable aggregation behaviour and remarkable thermostability, consistent with previously published results (44, 52). This approach of library generation enables the generation of Two-in-One antibodies targeting two unrelated proteins without additional engineering of the CDRs. In contrast to bispecific antibodies, which target one antigen with each Fab arm, HCP-LCE is capable of simultaneous binding two antigens with a single Fab, resulting in increased avidity.

An antibody subgroup that is also based on a common heavy chain are kappa-lambda (κλ) bodies. The κλ body platform uses a fixed heavy chain that is combined with a lambda or kappa light chain naïve or synthetic antibody repertoire and subsequently selected for target binding by phage display (59). HCP-LCE was generated according to a similar approach, with the difference that the Two-in-One antibody is not synthetic-, but rather derived from immune libraries.

The conventional, symmetric IgG architecture of HCP-LCE reduces the need for multi-step purification and allows for straight-forward manufacturing since the main challenge in producing bispecific antibodies arises from their heterogeneous structure (60). Like DutaFabs (18), HCP-LCE is able to simultaneously bind two target molecules with one Fab arm. The co-binding of two targets or epitopes on the same Fv fragment might enable unique mechanisms of action based on receptor clustering or on positioning proteins in functional distance. However, it is important to note that HCP-LCE does not contain two independent paratopes. BLI measurements indicated that exclusively the HCP-LCE heavy chain is responsible for PD-L1 binding, in contrast to EGFR binding involving overlapping heavy and light chain CDR residues. Binding to PD-L1 is not disrupted by the use of an unrelated light chain, suggesting that PD-L1 is targeted exclusively by the three heavy chain CDRs. However, both the heavy and light chains of HCP-LCE are required for EGFR binding, indicating that CDR residues of both antibody chains are responsible for binding. Nevertheless, the heavy chain CDR residues required for EGFR binding do not appear to overlap with the residues involved in PD-L1 binding, otherwise simultaneous binding would not be possible. To investigate the arrangement of both paratopes, co-crystallographic analysis would be of great interest.

Flow cytometric measurements demonstrated that HCP-LCE targets an epitope on domain II of EGFR, which is involved in receptor dimerization (48). Cetuximab and matuzumab, two EGFR-binding antibodies, are known to bind EGFR domain III, which together with domain I mediates EGF binding (53, 61). Although biolayer interferometric measurements confirmed that the EGFR domain II binder HCP-LCE does not block EGF binding, the Two-in-One antibody significantly inhibited EGFR downstream signaling, as demonstrated by analyzing AKT phosphorylation. Furthermore, HCP-LCE disrupted the PD-1/PD-L1 interaction by binding to an overlapping epitope with the therapeutic antibody durvalumab (44). Since the PD-1:PD-L1 axis is an immune checkpoint for NK cells and T cells, PD-L1 blockage may contribute to NK cell- and T cell-mediated killing (62–65).

Monoclonal antibody therapy is a treatment option for patients suffering from EGFR-related tumor burden (66). Since EGFR is natively expressed on epithelial cells in the skin and lung, the major side effect associated with treatment using EGFR targeting mAbs is skin toxicity, including skin rash, dry skin, hair growth disorders, and nail changes (67, 68). Koopmans and coworkers demonstrated an elevated tumor-specificity and tumor uptake by an EGFRxPD-L1 bispecific antibody (30). By simultaneous binding of EGFR and PD-L1, HCP-LCE might exhibit elevated tumor-selectivity, which could reduce side effects. This requires maximum discrimination between single-positive healthy cells and double-positive malignant cells. Moreover, due to the comparatively low affinity of HCP-LCE to EGFR (236 nM), increased EGFR cell expression, which is predominantly found on tumor cells (69), is required for targeting of the antibody. This could result in reduced on-target/off-tumor binding. In addition, the low binding affinity conceivably causes EGFR binding exclusively on cells that additionally express PD-L1 due to spatial proximity and local concentration. This concept has been described for bsAbs targeting tumor-specific receptors with high affinity on one arm and CD47 with lower affinity on the other arm (70–72). CD47 is ubiquitously expressed in human cells and has been found to be overexpressed in many tumors (73). The low-affinity binding to CD47 and the associated increased tumor selectivity results in a higher safety profile of the described bsAbs. Currently, four of these antibodies are in clinical or preclinical study (74). In the case of HCP-LCE, the antibody would inhibit PD-L1 in an EGFR-dependent manner, which could significantly reduce side effects.

HCP-LCE is a chimeric antibody consisting of chicken-derived VH and VL domains, grafted onto a human IgG1 scaffold. Starting from a heavy chain binder that was obtained by VH library screening likely facilitates the discovery of binders to a different target using a VL library of an immunized chicken. Unlike in mice, the light chain repertoire of chicken antibodies is generated *via* gene conversion (75). This might be one reason for chicken-derived antibodies being suitable for the generation of light chain binders. In regard to immunogenicity, humanization of the Two-in-One antibody is essential for potential therapeutic applications. Our group recently demonstrated an effective approach to humanize avian-derived antibodies based on Vernier residue randomization and high throughput screening (76) that could be applied for this purpose but is beyond the scope of this proof-of-concept study.

Taken together, we present a straightforward method for the isolation of chicken-derived Two-in-One antibodies without CDR engineering by combining the heavy chain of an anti-PD-L1 common light chain antibody with an anti-EGFR immune light chain library and YSD screening. The resulting antibody HCP-LCE simultaneously targets EGFR and PD-L1 at the same Fv fragment while exhibiting favorable biophysical properties and aggregation behaviour. The Two-in-One antibody is able to inhibit EGFR signaling by binding to dimerization domain II and can also block the PD-1/PD-L1 interaction. Furthermore, HCP-LCE demonstrated specific cellular binding properties on EGFR/PD-L1 double-positive tumor cells. To our knowledge, this represents the first Two-in-One antibody without CDR engineering that simultaneously targets two antigens with one Fab fragment.

## Material and Methods

### Plasmids and Yeast Strains

For yeast surface display, pYD_1_-derived vectors (Yeast Display Vector Kit, version D, #V835-01, Thermo Fisher Scientific) were used. The heavy chain encoding plasmid contained the *AGA2* signal peptide, followed by the ICI2 (44) VH-CH_1_ sequence and the *AGA2* gene, a tryptophan auxotrophic marker as well as an ampicillin resistance. The light chain plasmid encoded an αMFpp8 signal sequence, followed by the VL-CLλ sequences, a leucine auxotrophic marker and a kanamycin resistance gene. Gene expression of either plasmid was controlled *via* the galactose-inducible promotor (*GAL_1_*). For soluble expression of full-length chimeric antibodies, pTT5-derived vectors (47) were used, encoding either the heavy or light chain constant domains. Bispecific variants were expressed using pTT5-derived vectors encoding the full-length chimeric antibody with either a knob or hole mutation (10) within the CH_3_ sequence, and a C-terminal His- or Twin-StrepII-Tag, respectively. For the one-armed variant, a pTT5-derived vector encoding the Hinge-CH_2_-CH_3_ domain with the hole mutation and a C-terminal Twin-StrepII-Tag was utilized.

The Saccharomyces cerevisiae strains EBY100 [MATa URA3-52 trp1 leu2Δ1 his3Δ200 pep4::HIS3 prb1Δ1.6R can1 GAL (pIU211:URA3)] (Thermo Fisher Scientific) and BJ5464 (MATα URA3-52 trp1 leu2Δ1his3Δ200 pep4::HIS3 prb1Δ1.6R can1 GAL) (American Type Culture Collection) were transformed with the plasmids harbouring the heavy chain and light chain genes for Fab display, respectively. Yeast strains were cultivated in YPD medium composed of 20 g/L peptone/casein, 20 g/L glucose and 10 g/L yeast extract. Cultivation of haploid and diploid yeasts in SD-CAA and SG-CAA media was performed as described previously (77).

### Library Generation and Sorting

For yeast library generation, the VH-CH_1_ fragment of the anti-PD-L1 antibody ICI2 (44) was combined with an anti-EGFR VL-CLλ library (44). The ICI2 VH gene was amplified by PCR using Q5 polymerase (NEB) and the heavy chain pYD_1_ vector was linearized utilizing NheI HF and BamHI-HF (NEB) according to the manufacturer´s protocol. Homologous recombination of ICI2 VH gene into pYD_1_ was conducted in EBY100 yeast cells according to the protocol described by Benatuil et al. (78).

Generation of the utilized anti-EGFR VL-CLλ yeast library was described by Grzeschik et al. (35). To combine the light chain diversity with the common ICI2 heavy chain for subsequent Fab display, yeast mating was performed as described before (77).

For library sorting, cells of the diploid yeast library were grown overnight in SD-Trp-Leu medium at 30°C and 120 rpm. The next day, cells were harvested by centrifugation and used to inoculate SG-Trp-Leu medium at an OD_600_ of 1.0 and incubated overnight at 30°C and 120 rpm. Cells were harvested by centrifugation, washed once with PBS-B [PBS + 0.1% (w/v) BSA] and incubated with 250 nM EGFR-ECD-Fc chimera (R&D Systems) for 30 min on ice. After washing once with PBS-B, cells were incubated with a goat anti-human-Lambda Alexa Fluor 647 F(ab´)2 antibody (SouthernBiotech, diluted 1:75) to detect Fab surface presentation, and a goat anti-human IgG-Fc-PE conjugate (Fisher Scientific, diluted 1:50) to detect target binding for 15 min on ice. Following another washing step, cells were screened by FACS using a Sony SH800S.

### Reformatting, Expression and Purification of Full-Length, One-Armed and Bispecific Antibodies

Plasmid isolation from yeast cells was performed using the Zymoprep Yeast Plasmid Miniprep Kit (Zymo Reasearch) according to the manufacturer`s protocol. Isolated plasmids were transformed into *E. coli* XL1-Blue and sequenced at Microsynth Seqlab (Göttingen). The resulting VL gene was amplified by PCR using Q5 polymerase (NEB) according to the manufacturer´s protocol, incorporating *SapI* sites to enable subsequent Golden Gate cloning into pTT5-derived vectors as described previously (47). For soluble expression, Expi293F (Thermo Fisher, A14527) cells were transiently transfected following the manufacturer`s protocol. Cells were cultivated in Expi293 Expression Medium (Thermo Fisher) at 37°C and 8.0% CO_2_ at 110 rpm. For purification of full-length antibodies, sterile-filtered cell culture supernatant was applied to a Protein A HP column (GE Healthcare) five days after transfection using an ÄKTA pure system (GE Healthcare). One-armed and bispecific molecules were captured by IMAC (HisTrap HP, GE Healthcare), followed by Strep-Tactin XT affinity chromatography according to the manufacturer´s protocol. Buffer exchange against PBS was performed using a HiTrap Desalting column (GE Healthcare).

### Epitope Mapping on the Subdomain Level *via* YSD

YSD-based epitope mapping was performed using yeast cells displaying six different truncated versions of EGFR-ECD (amino acids 1-124, 1-176, 1-294, 273-621, 294-543 and 475-621), as described previously (51, 52). Cells were harvested by centrifugation, washed once with PBS-B and incubated with 200 nM HCP-LCE for 30 min on ice. Surface presentation was verified using a biotinylated anti-c-myc antibody (Miltenyi Biotech, diluted 1:75) and Streptavidin APC (Thermo Fisher, diluted 1:75). Separately, antibody binding was verified by an anti-human IgG Fc PE-conjugated antibody (Fisher Scientific, diluted 1:50). Cells were analyzed by flow cytometry using a SH800S (Sony Biotechnology).

### Affinity Determination, Receptor-Ligand Competition and Simultaneous Binding Assay *via* Biolayer Interferometry

For affinity determination of chimeric antibodies, anti-human IgG-Fc capture (AHC) biosensors were equilibrated in PBS pH 7.4 for 10 min and subsequently loaded with 10 µg/ml of the antibody of interest until a layer thickness of 1 nm was reached. All following steps were performed using kinetics buffer (KB, Sartorius). Association was measured for 600 s using varying concentrations of EGFR-ECD or PD-L1-ECD (produced in-house) ranging from 7.8 nM to 500 nM followed by dissociation for 600 s. KB was used as a negative control. Binding kinetics were determined based on Savitzky-Golay filtering and a 1:1 Langmuir binding model.

For the EGF competition assay, AHC biosensors were loaded with 10 µg/ml of HCP-LCE until a layer thickness of 1 nm was reached. Subsequently, 250 nM EGFR-ECD pre-incubated with either 0 nM, 250 nM or 1000 nM EGF was applied for 600 s.

For the PD-1 competition assay, anti-human Fab-CH_1_ 2nd Generation (FAB2G) biosensors were loaded with 10 µg/ml of HCP-LCE until a layer thickness of 1 nm was reached. Subsequently, 250 nM PD-L1-ECD pre-incubated with either 0 nM, 250 nM or 1000 nM PD-1 was applied for 600 s.

For the simultaneous binding assay, AHC biosensors were loaded with 10 µg/ml of oaHCP-LCE until a layer thickness of 1 nm was reached. After measurement of the association to 250 nM antigen 1 for 300 s, association to 250 nM antigen 2 was determined for 300 s. As controls, oaHCP-LCE was incubated with antigen 1 or PBS only. EGFR-ECD and PD-L1-ECD were used as antigens and the order of association was analyzed in both settings.

All measurements were performed using the Octet RED96 system (FortéBio, Molecular Devices) at 30°C and 1000 rpm.

### NanoDSF and Size Exclusion Chromatography

Thermal stability of produced antibodies was characterized by nano differential scanning fluorimetry (NanoDSF) using the Prometheus NT.48 Protein Stability Instrument (NanoTemper Technologies). Tryptophan fluorescence of a 0.5 mg/mL protein solution was measured at 350 and 330 nm applying a temperature gradient from 20°C to 95°C with a temperature slope of 1°C/min. T_M_ values were defined as the first maxima of the ratios of the first derivative of fluorescence at 330 nm and 350 nm.

Size exclusion chromatography (SEC) using TSKgel SuperSW3000 column (Tosoh Bioscience) together with 1260 Infinity chromatography system (Agilent Technologies) was utilized to analyze the aggregation behaviour of antibodies. Chromatography was performed at a flow rate of 0.35 mL/min for 20 min, and protein elution was detected by measuring absorbance at 280 nm.

### Cultivation of A431 and A549 Cells

A431 human epidermoid carcinoma cells (ATCC^®^ CRL-1555™) and A549 human epithelial lung carcinoma cells (DSMZ ACC 107) were cultured in Dulbecco´s Modified Eagle Medium (DMEM, Thermo Fisher), supplemented with 10% fetal bovine serum (FBS) superior (Merck Millipore) and 1% Penicillin-Streptomycin (Sigma Aldrich). Cells were cultured in T75 cell culture flasks at 37°C in a humidified atmosphere with 5% CO_2_ and passaged every three to four days after reaching 80% confluence.

### EC50 Determination

Cellular binding of the produced antibodies was determined by affinity titration using EGFR/PD-L1 double positive A431 cells. EGFR/PD-L1 double negative HEK cells were used to analyze unspecific cell binding. To this end, 10^5^ cells/well were seeded in 96-well plates, washed with PBS-F [PBS + 2% (w/v) FBS] and subsequently incubated with the respective antibody in varying concentrations (500 nM to 0.24 nM in a two-fold serial dilution) for 30 min on ice. Following another washing step, anti-human IgG-Fc PE-conjugated antibody was applied for 20 min. After washing, mean fluorescence was determined by flow cytometry using a CytoFLEX S (Beckman Coulter) and plotted against logarithmic antibody concentration. The resulting curves were fitted with a variable slope four-parameter fit using GraphPad Prism. All measurements were performed in triplicates, and the experiments were repeated at least three times, yielding comparable results.

### AKT Pathway Signaling Assay

Two days prior to the assay, A549 cells were seeded onto sterile 48-well cell culture plates at a cell density of 10^5^ cells/well. The following day, cells were serum-starved in DMEM medium overnight. Subsequently, cells were pre-incubated with the desired antibody concentration for 1 h, followed by stimulation with 20 ng/mL rhEGF for 10 min at 37°C. Following stimulation, cells were quickly rinsed with pre-chilled PBS and lysed using Complete Lysis Buffer. For AKT phosphorylation analysis, the cell lysates were analysed using the AKT Signaling Whole Cell Lysate Kit (MesoScale Discovery) according to the manufacturer’s protocol. The electrochemiluminescence (ECL) values were plotted using GraphPad Prism.

### Cell-Based PD-1/PD-L1 Blockage Reporter Assay

For the cell-based PD-1/PD-L1 blockade assay, the Promega PD- 1/PD-L1 Blockade Bioassay (J1250) was used according to the manufacturer´s instructions. Antibodies of interest were tested at a 3-fold dilution series, ranging from 2 µM to 0.3 nM for HCP-LCE, ICI2_H2 and SEB7xICI2_H2 and from 222.2 nM to 0.1 nM for Durvalumab. SEB7 was used as a control at a concentration of 2 µM. After incubation at 37°C and 5% CO_2_ for six hours, luciferase activity was measured and plotted against logarithmic antibody concentration. The resulting curves were fitted utilizing a variable slope four-parameter fit.

## Data Availability Statement

The original contributions presented in the study are included in the article/**Supplementary Material**. Further inquiries can be directed to the corresponding author.

## Author Contributions

JH, JB and HK conceived and designed the majority of experiments. JH and SC performed experiments. JH, JB, SC and HK analyzed the data. MU, BH and JG gave scientific advice. JH, JB and HK wrote the manuscript. All authors contributed to the article and approved the manuscript.

## Funding

This work was supported by the Ferring Darmstadt Laboratories at Technical University of Darmstadt and by the department of GPRD at Ferring Holding S.A., Saint-Prex. The funders had no role in study design, data collection and analysis, interpretation of data, decision to publish, or preparation of the manuscript. All authors declare no other competing interests.

## Conflict of Interest

BH and JG are employees of Ferring Pharmaceuticals. JB, SC and MU were employed by TU Darmstadt in frame of a collaboration project with Ferring Pharmaceuticals. HK, JH, JB, SC and MU are inventors of a patent related to the Two-in-One antibody HCP-LCE (EP22159491.4).

The remaining authors declare that the research was conducted in the absence of any commercial or financial relationship that could be construed as a potential conflict of interest.

## Publisher’s Note

All claims expressed in this article are solely those of the authors and do not necessarily represent those of their affiliated organizations, or those of the publisher, the editors and the reviewers. Any product that may be evaluated in this article, or claim that may be made by its manufacturer, is not guaranteed or endorsed by the publisher.
