# Supplementary material for: A Generic Strategy to Generate Bifunctional Two-in-One Antibodies by Chicken Immunization
Source: Front Immunol. 2022 Apr 11;13:888838. doi: 10.3389/fimmu.2022.888838 (PMC9036444; doi:10.3389/fimmu.2022.888838)
Supplement: Supplementary file 1 [file DataSheet_1.docx]

Supplementary Material


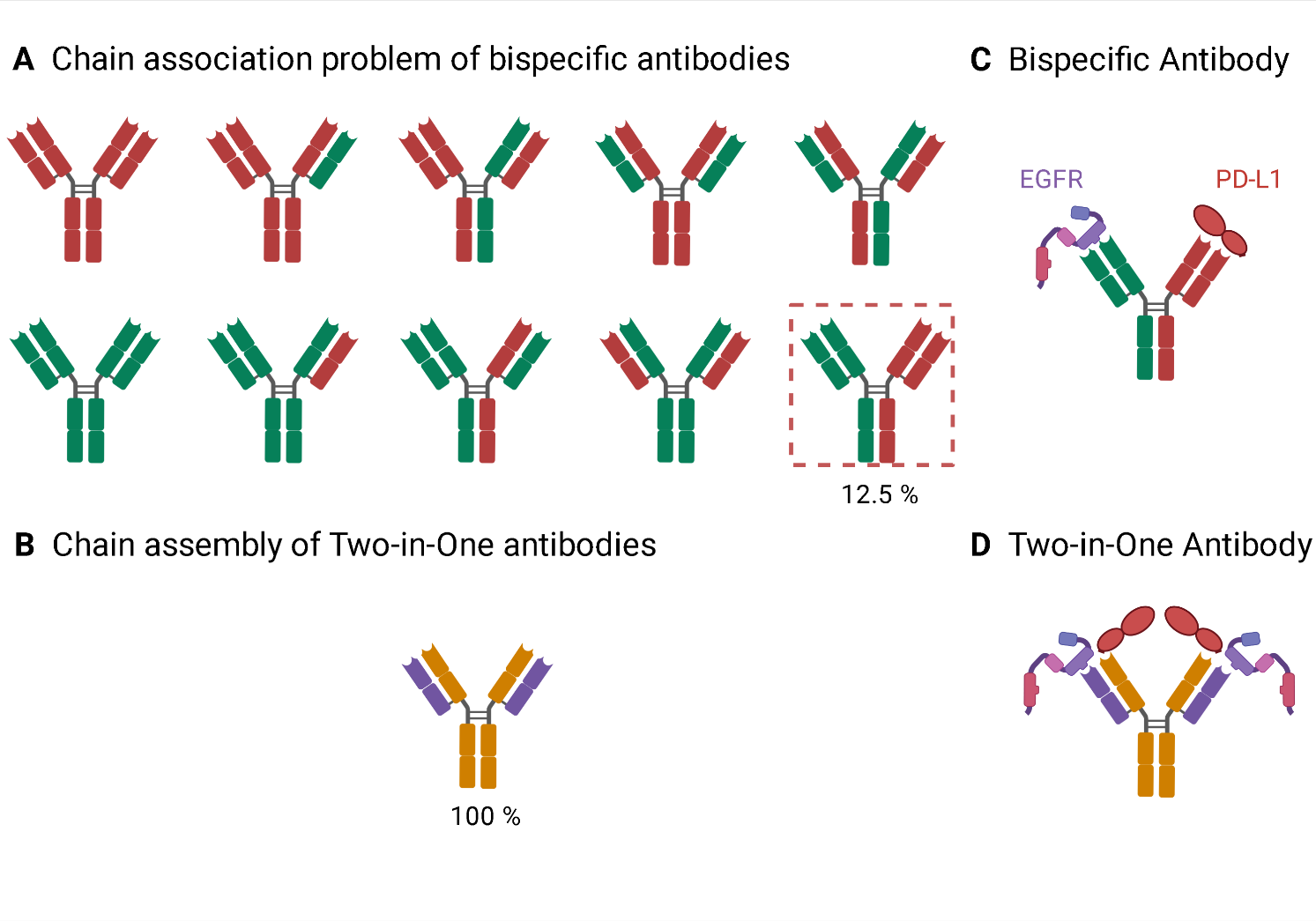


**Supplementary Figure 1.** Chain assembly of bispecific antibodies. A) Possible heavy and light chain pairing combinations of bispecific antibodies. The correctly paired variant is marked in red. B) For Two-in-One antibodies, there is only one possibility of chain assembly. C) A bispecific antibody binds one antigen with each Fab fragment. D) Two-in-One antibodies target two antigens with each Fab arm.


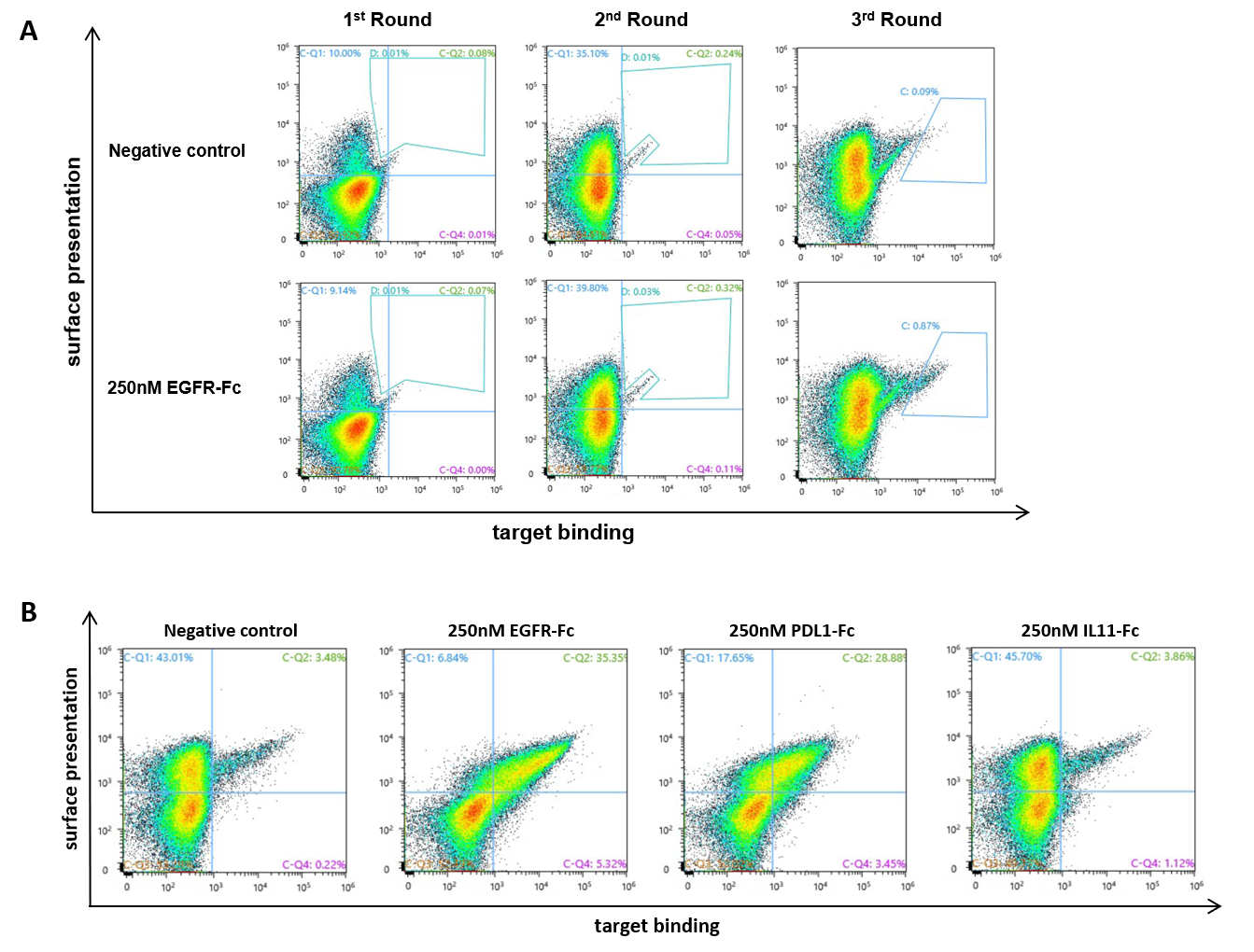


**Supplementary Figure 2.** Isolation of HCP-LCE. A) Sorting of the diploid common heavy chain yeast library. Surface presentation is depicted on the y-axis utilizing the anti-human lambda chain antibody AF647 labelled, while EGFR-Fc binding is shown on the x-axis using the anti-human Fc-PE antibody. B) Flow cytometric analysis of the isolated yeast population after three consecutive rounds of FACS screening. Surface presentation is depicted on the y-axis utilizing the anti-human lambda chain antibody AF647 labelled, while EGFR‑Fc, PD-L1-Fc and IL11-Fc binding is shown on the x-axis using the anti-human Fc-PE antibody.


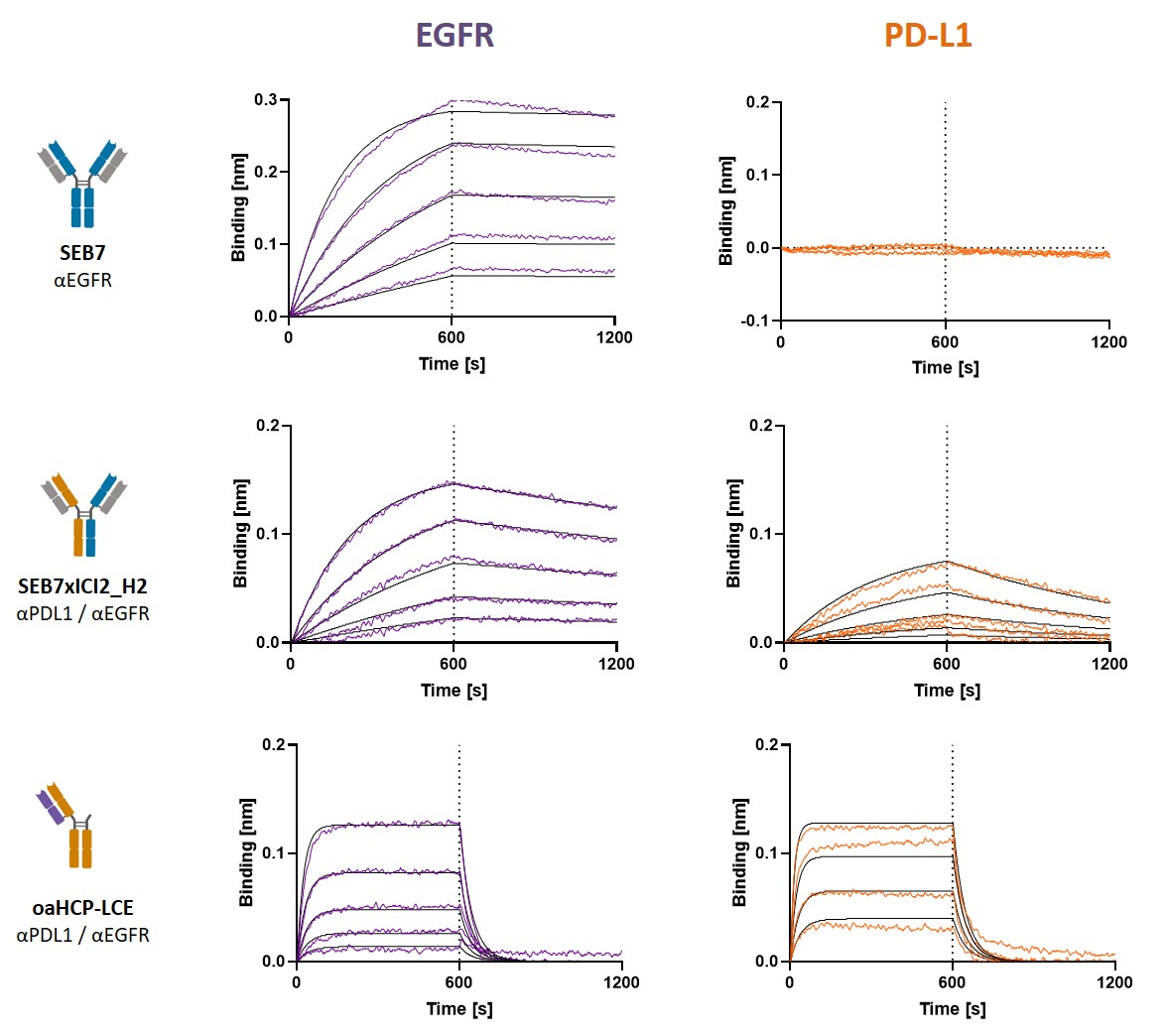


**Supplementary Figure 3.** Affinity measurements. Binding kinetics of SEB7, SEB7xICI2_H2 and oaHCP-LCE to EGFR and PD-L1.


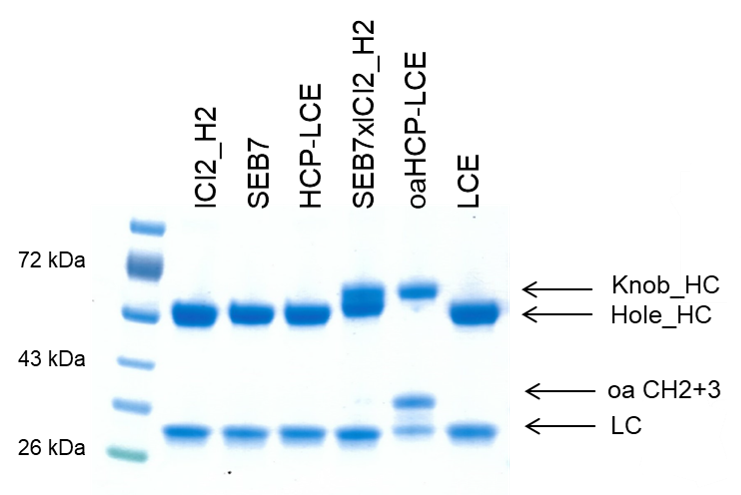


**Supplementary Figure 4.** SDS-PAGE analysis of ICI2_H2, SEB7, HCP-LCE, SEB7xICI2_H2, oaHCP-LCE and LCE under reducing conditions revealed high purity and the expected molecular weights.


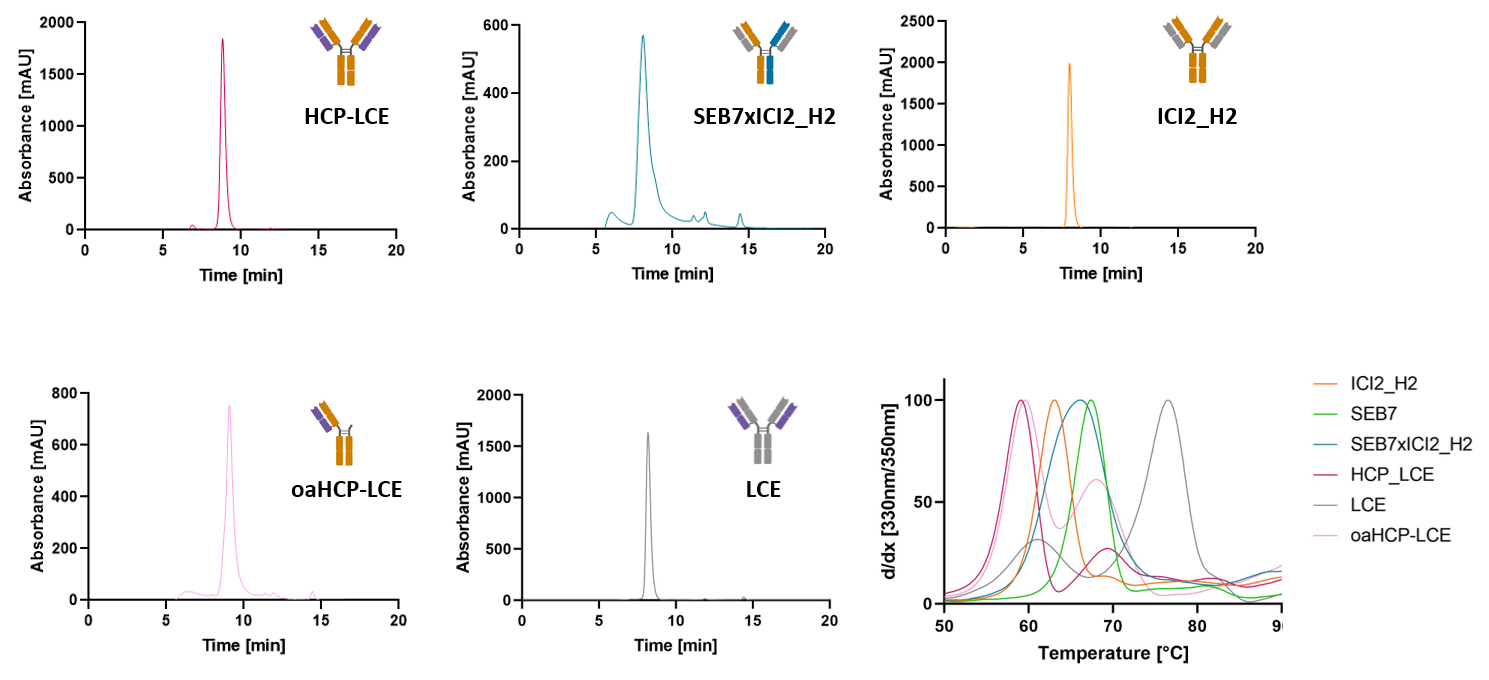


**Supplementary Figure 5.** Characterization of HCP-LCE (pink), SEB7xICI2_H2 (blue), ICI2_H2 (orange), oaHCP-LCE (light pink) and LCE (grey). SEC profiles and NanoDSF measured melting temperatures.


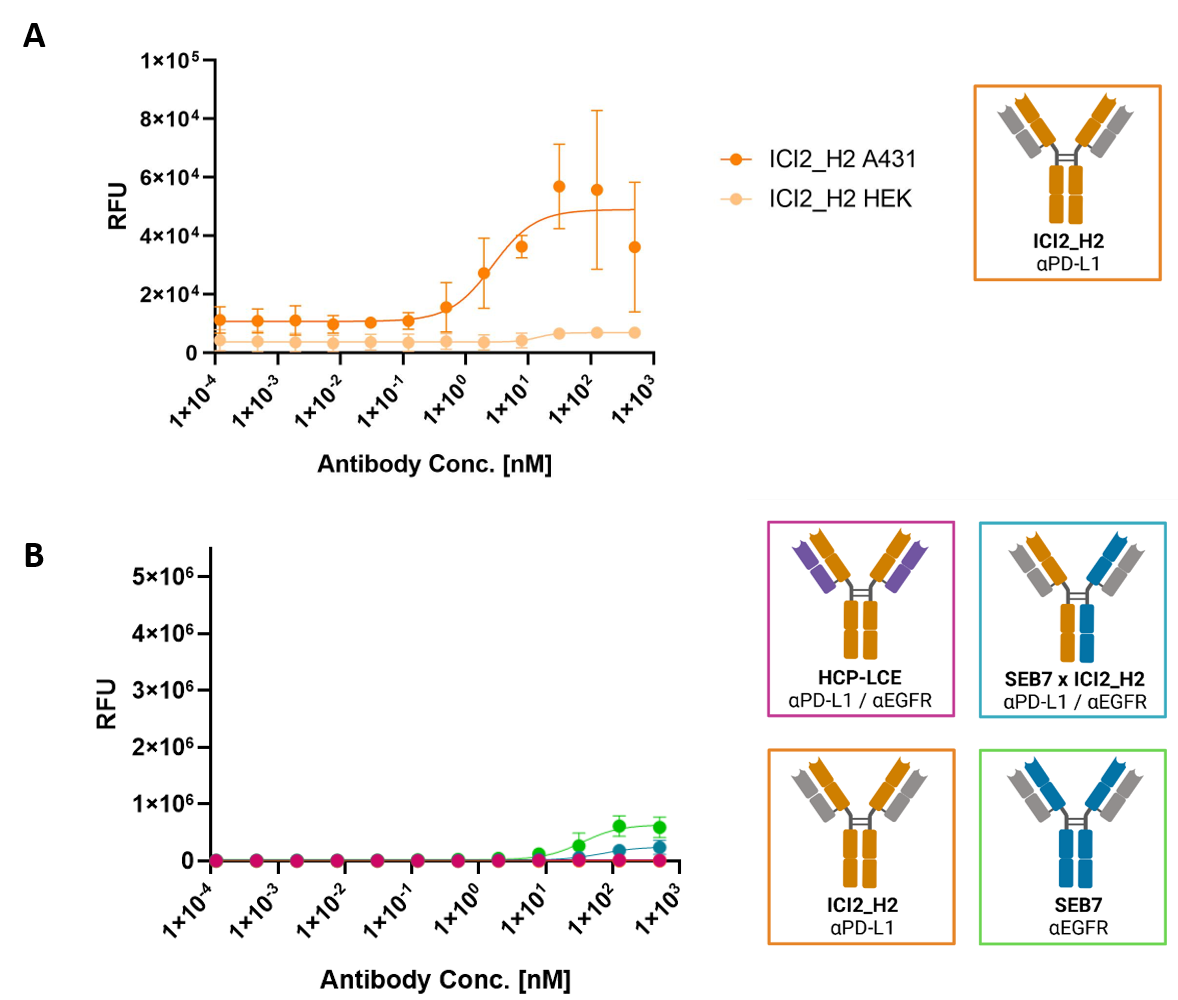


**Supplementary Figure 6.** Cellular binding on A431 cells and HEK cells. A) Cell titration of ICI2_H2 on EGFR/PD-L1 double positive A431 cells (orange) and on EGFR/PD-L1 double negative HEK cells (light orange). A variable slope four-parameter fit was utilized to fit the resulting curves. B) Cell titration of HCP-LCE, SEB7xICI2_H2, ICI2_H2 and SEB7 on EGFR/PD-L1 double negative HEK cells. The y-axis is chosen as in figure 6 for comparability of the graphs. A variable slope four-parameter fit was utilized to fit the resulting curves. The assay was repeated twice, yielding comparable results.
